# Microfluidic droplet generation based on non-embedded co-flow-focusing using 3D printed nozzle

**DOI:** 10.1038/s41598-020-77836-y

**Published:** 2020-12-10

**Authors:** Adrien Dewandre, Javier Rivero-Rodriguez, Youen Vitry, Benjamin Sobac, Benoit Scheid

**Affiliations:** TIPs Lab, Université libre de Bruxelles, Brussels, 1050 Belgium

**Keywords:** Microfluidics, Fluid dynamics

## Abstract

Most commercial microfluidic droplet generators rely on the planar flow-focusing configuration implemented in polymer or glass chips. The planar geometry, however, suffers from many limitations and drawbacks, such as the need of specific coatings or the use of dedicated surfactants, depending on the fluids in play. On the contrary, and thanks to their axisymmetric geometry, glass capillary-based droplet generators are a priori not fluid-dependent. Nevertheless, they have never reached the market because their assembly requires fastidious and not scalable fabrication techniques. Here we present a new device, called Raydrop, based on the alignment of two capillaries immersed in a pressurized chamber containing the continuous phase. The dispersed phase exits one of the capillaries through a 3D-printed nozzle placed in front of the extraction capillary for collecting the droplets. This non-embedded implementation of an axisymmetric flow-focusing is referred to non-embedded co-flow-focusing configuration. Experimental results demonstrate the universality of the device in terms of the variety of fluids that can be emulsified, as well as the range of droplet radii that can be obtained, without neither the need of surfactant nor coating. Additionally, numerical computations of the Navier-Stokes equations based on the quasi-steadiness assumption allow to provide an explanation to the underlying mechanism behind the drop formation and the mechanism of the dripping to jetting transition. Excellent predictions were also obtained for the droplet radius, as well as for the dripping-jetting transition, when varying the geometrical and fluid parameters, showing the ability of this configuration to enventually enhance the dripping regime. The monodispersity ensured by the dripping regime, the robustness of the fabrication technique, the optimization capabilities from the numerical modelling and the universality of the configuration confer to the Raydrop technology a very high potential in the race towards high-throughput droplet generation processes.

## Introduction

**Figure 1:**
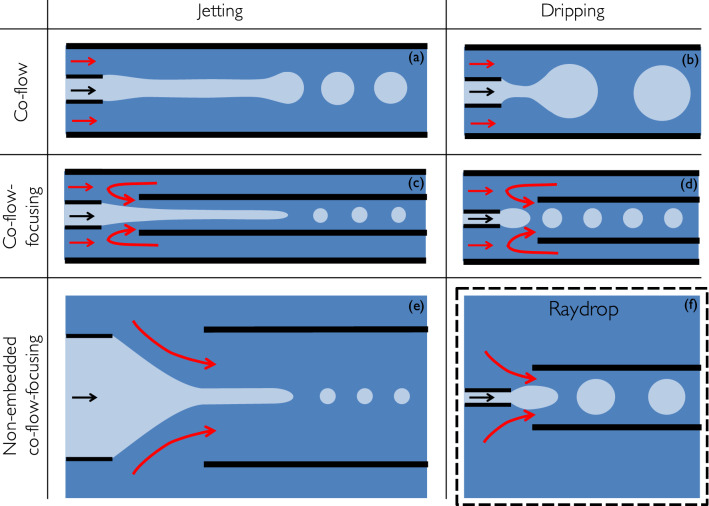
Capillary-based axisymetric designs of droplet generators. (**a**) and (**b**) jetting and dripping of a co-flow, respectively^20^; (**c**) and (**d**) jetting and dripping of a co-flow-focusing in an embedded geometry, respectively^22^; (**e**) co-flow-focusing in a non-embedded geometry, enforcing the jetting regime^25^, also referred to as tip-streaming; (**f**) Raydrop: co-flow-focusing in a non-embedded geometry, enforcing the dripping regime. All solid black lines represent capillary walls.

In recent years, droplet microfluidics^1–5^ has become an important tool for many different applications, including fundamental studies on emulsification^6^, crystallization^7^ and chemical reaction^8^, temperature-controlled tensiometry^9^, molecules encapsulation^10^, particle synthesis^11^, biochemical assays^12^, immunoassays^13^, digital PCR^14^, directed evolution^15^, drug discovery^16^, single-cell analysis^17^ or cell and gene manipulations^18,19^. Nowadays, all the commercially available and most of lab-made droplet generators are based on a flow-focusing technology implemented in rectangular microchannels fabricated by lithography, and made of polydimethylsiloxane (PDMS), polymers or glass. However, this planar configuration has many limitations, mostly due to the contact between the walls of the microchannels and both phases at the junction, requiring laborious and often ephemeral wettability treatments of these walls. On the contrary, due to their axisymmetric configuration, glass capillary systems do not have this drawback since the dispersed phase is never in contact with the walls of the outer capillary^20^. Yet their widespread use is limited by the difficulty to implement this technology in an easy-to-use device. And even if co-flow configurations have been set up using commercially available components^21^ (see Fig. 1a,b), these devices produce large droplets ($>100~\upmu$m) at low throughput and are unable to generate small ($< 100~\upmu$m) and monodisperse droplets at high throughput ($>1$ kHz), as realised in planar flow focusing configuration. Furthermore, the centering of the capillary into an outer flow capillary is challenging. Even though the flow focusing configuration has also been designed using glass capillaries by inserting two circular capillaries into a square outer flow capillary, which greatly simplifies centering of the capillaries^22^ (see Fig. 1c,d), three main limitations remain. Firstly, the restrictions necessary to obtain a focusing effect at the tip of the inner injection and extraction capillaries are obtained by pulling and breaking a heated glass capillary, a very art-dependent technique limiting the use of this design in large-scale microfluidic applications. Secondly, the confined space for flowing the continuous phase around the inner capillaries limits its flow rate, hence the throughput, in the droplet generation process, particularly with highly viscous fluids. And finally, the interfacing with the tubing carrying the fluids is typically realised by gluing hypodermic needles over the embedded capillaries previously glued on a glass slide. This fabrication process not only limits the reuse of the device because it can not be disassembled and properly cleaned, but also make it not suitable for large-scale production. Alternative procedures have been developed to improve the usability of embedded capillary devices^23,24^, but none can overcome all these limitations.

A configuration proposed by Evangelio *et al.*^25^ has revealed a promising alternative by placing the extraction tube without any surrounding confinement in front of the injection tube (see Fig. 1e). The continuous phase is therefore accelerated to the extraction tube, thus creating a pressure drop, similar to a Venturi tube, even though viscous forces are dominant in this case. The authors have exploited this pressure drop to create a stretched jet of the dispersed phase, that further destabilizes into micro-bubbles or droplets, a mechanism which is referred to as the “tip streaming”. However, their system exclusively works in the jetting regime, which does not intrinsically guarantee the droplet monodispersity associated to the dripping regime, as realised in embedded glass capillaries^22^, even though the polydispersity of droplet sizes obtained with the tip streaming configuration could be lower than the one obtained with other jetting mode configurations. In this work, we consider a new configuration based on the configuration proposed by Evangelio *et al.*^25^ but in which the dripping regime is enforced.

**Figure 2:**
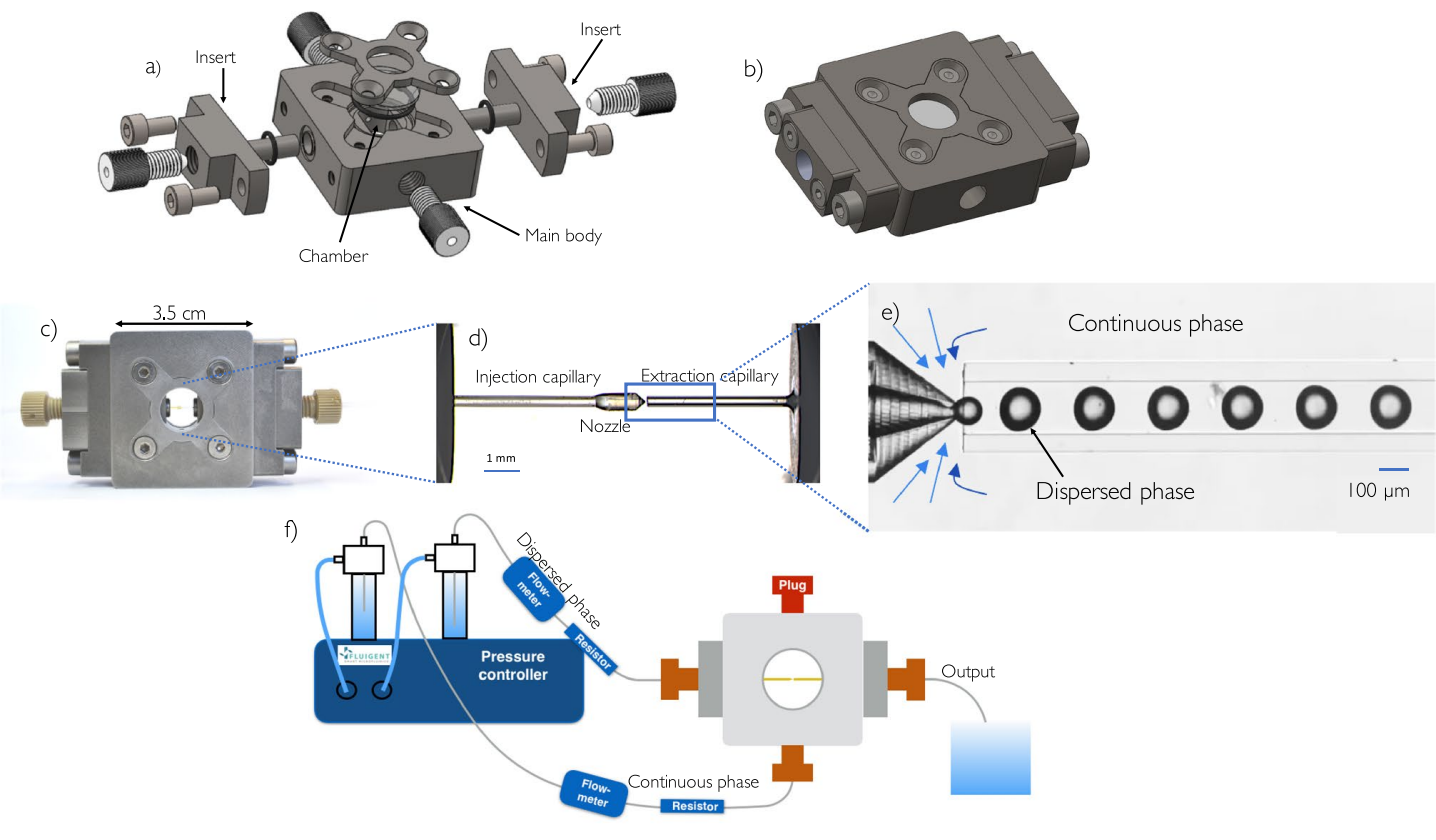
(**a**) Exploded view and (**b**) assembled view of the Raydrop. (**c**) The Raydrop with the injection and extraction glass capillaries. (**d**) Zoom through the top window of the two capillaries aligned in the chamber filled with the continuous phase. (**e**) Zoom on the droplet generation area. The 3D-printed nozzle connected to the injection capillary carries the dispersed phase while the extraction capillary collects droplets of the dispersed phase entrained by the continuous phase. At the entrance of the extraction capillary, the continuous phase is dramatically accelerated because of the change of section and squeezes the dispersed phase, resulting in the droplets formation. (**f**) Setup used for the production of droplets using the Raydrop. The flows are controlled using a pressure controller and the flow rates are measured using flow-meters. The optical equipment allowing the observation of droplets by transmission through the windows, i.e. light source, microscope and high-speed camera, are not shown.

Our device relies on the alignment of two glass capillaries inside a pressurised chamber, similarly to Evangelio *et al.*^25^. However, while their configuration relies on an inner diameter of the injection capillary larger than the one of the extraction capillary, such as it can stably operate in the jetting mode only, our configuration can operate in the dripping mode also because the injection capillary has a smaller diameter than the extraction one. This configuration could only be obtained thanks to the proper combination of cutting-edge machining and 3D printing techniques to fabricate a micro-nozzle connected at the tip of the injection capillary (see Fig. 2d) and align it with the extraction capillary, enforcing the dripping of small droplets as in Utada *et al.*^22^. This non-embedded design presents both the characteristics of a co-flow (axisymetric geometry) and a flow-focusing (dramatic local accelerations of the continuous phase), and is thereby called *non-embedded* *co-flow-focusing*. As illustrated in Fig. 1f, this configuration fills the gap in the design array of microfluidic droplet generators. The technological breakthrough of this design is two-folds. Firstly, it enables the generation of monodisperse droplets, intrinsic to the dripping regime, at high throughput and for a wide variety of fluids, thanks to the fact that the continuous phase is not confined before entering the extraction capillary, allowing for flushing very viscous continuous phases. Secondly, it takes benefit of specific fabrication techniques and materials compatible with a large-scale production of the device, while ensuring a very precise and reproducible alignment of the two capillaries in the chamber. Additionally, the device is made plug-and-play thanks to the standard connections and the possibility to easily assemble and disassemble all parts for cleaning.

Besides the axisymmetric configuration proposed by Evangelio *et al.*^25^ in the jetting mode, one should mention the recent experiment performed by Cruz-Mazo *et al.*^26^ that exploits the axisymmetric flow-focusing in the dripping mode. To our knowledge, this is the closest configuration to the one of the Raydrop. Yet these authors used exclusively gas as continuous phase and found that the liquid drop size is independent of the flow rate of the dispersed phase, a feature that suggests a quasi-static behavior, as exploited in the present work.

The ultimate aim in droplet generation being to predict the size of droplets, initial theoretical works have relied on linear stability analysis of the co-flow configuration and found that the dripping and jetting regimes exhibit the main features of absolutely and convectively unstable flows, respectively^20,27^. However, Cordero *et al.*^28^ have shown that the frequency selection in the dripping regime is not ruled by the absolute frequency predicted by the stability analysis. Thus, recognizing the nonlinear behaviour of droplet formation in the dripping regime, many numerical studies have been performed in the co-flow configuration using the Navier-Stokes equations together with diverse methods to describe the interfacial dynamics, as for instance the finite volume method combined with continuous-surface-force method^29^, front tracking method^30^ or level-set method^31^. For the flow-focusing configuration in a cross-junction microchannel, one can mention the diffuse interface or phase-field method^32^, or the work by Wu *et al.*^33^ using three-dimensional lattice Boltzmann simulations. Nevertheless, computationally intensive three-dimensional simulations and the number of geometrical parameters involved in a planar flow-focusing configuration have prevented so far systematic and complete numerical analysis of the drop formation in such a configuration. Using Finite Element Method (FEM) with adaptive meshing in a diffuse-interface framework, Zhou *et al.*^34^ have simulated an axisymmetric flow-focusing configuration, yet involving a large number of geometrical parameters. The non-embedded co-flow-focusing (see Fig. 1e,f) presented here reduces the configuration to the minimum number of independent parameters, as compared to planar flow-focusing or embedded co-flow-focusing devices (see Fig. 1c,d), then making more accessible an exhaustive parametric analysis. Yet the task is still huge and some additional simplifications are needed.

Several authors have recognised the quasi-static behavior of the dripping mode. For instance, Garstecki *et al.*^35^ claimed that the quasi-static character of their data collapse forms the basis for controlled, high-throughput generation of monodisperse fluid dispersions. In the step-emulsification configuration, Li *et al.*^36^ solved the quasi-static shape of an elongated drop using the 1D equations of the Hele-Shaw cell. Inspired by these quasi-static behaviors, and thinking about the co-flow-focusing configuration, which is intrinsically axisymmetric, the analogy with the pendant droplet becomes evident. In this context, the quasi-static assumption applies, provided the liquid is injected at a sufficiently low flow rate into the droplet in formation. Boucher *et al.*^37^ for instance have shown that the dripping of a pendant droplet coincides with a folding bifurcation, indicating the maximum drop volume above which surface tension cannot sustain the drop weight anymore. But gravity can obviously be replaced by other forces, such as surface forces^38^, or electric forces^39^. We claim in this paper that the quasi-static approach can be adopted analogously to the pendant drop method, provided the flow rate of the dispersed phase is small enough and that the role of gravity is essentially played by the hydrodynamic forces acting on both phases and originated by the imposed continuous flow rate. As mentioned above, this situation has been experimentally considered by Cruz-Mazo *et al.*^26^, but only in the case of air as continuous phase. We here consider all situations in the quasi-static approach, from zero viscosity ratio corresponding to inviscid droplets, to infinite viscosity ratio corresponding to highly viscous droplets.

In the context of simulating multi-components flows, interface tracking methods, such as FEM with moving mesh algorithm, are very accurate for simulating the onset of droplet break-up but have difficulties in simulating through and past the transitions as it requires cut-and-connect procedures for the mesh^40^. Assuming that the volume of the dispersed phase preceding the pinch-off corresponds exactly to the volume of the droplet after break-up, there is in fact no need to simulate the dynamics beyond the transition if one only wants to determine the volume of the generated droplet. Following this approach, Martinez-Calvo *et al.*^41^ have applied FEM to investigate the break-up of liquid threads and determine the volume of satellite droplets, with an impeccable accuracy. They have solved the Navier-Stokes equations using the finite element software Comsol, and more precisely, the weak form Partial Differential Equation (PDE) and the Arbitrary Lagrangian-Eulerian (ALE) modules. Note that the jet breaking is intrinsically transient as it results from the Rayleigh-Plateau instability but the droplet generation, as mentioned above, can be quasi-static in the limit of low dispersed flow rate. Van Brummelen *et al.*^42^ having shown that solving the transient Navier-Stokes equation to obtain the steady solution is often inefficient, Rivero-Rodriguez *et al.*^43^ have developed the Boundary Arbitrary Lagrangian-Eulerian (BALE) method to facilitate the solution of steady Navier-Stokes equations with free surfaces. Rivero-Rodriguez and Scheid^44,45^ have then applied this method to study the dynamics of deformable and off-centered bubbles in microchannels, allowing for exhaustive parametric analysis, taking the advantage of continuation methods suitable for tracking stationary solutions. The same approaches are undertaken in the present paper in which the unprecedented quasi-static simplification allows for an extended parametric analysis in the context of flow-focusing.

After detailing the Raydrop configuration in section Materials and methods, we show in section Experiments the experimental measurements of droplet size and frequency for two different geometries, with the aim to identify experimentally the dripping to jetting transition when increasing both the dispersed and the continuous flow rates. Section Modelling is dedicated to the model description and validation in regards to the experimental results, and especially the relevance of the quasi-static approach for determining the droplet radius in the dripping regime in the limit of small dispersed flow rate. Section Parametric analysis focuses on the quasi-static approach, first for inviscid droplets, then for viscous ones, followed by a parametric analysis of the geometrical parameters, and a discussion on the role of inertia for properly predicting the dripping to jetting transition when increasing the continuous flow rate. General conclusions and perspectives are given in the last section, Discussions and Conclusions.

## Materials and methods

The droplet generator Raydrop is made of a metallic chamber filled with the continuous phase, in which two inserts supporting the glass capillaries are introduced on the lateral sides in such a way that the capillaries are perfectly aligned and almost in contact at the centre of the chamber, as shown in Fig. 2.

Two glass windows on the top and bottom faces of the device close the chamber and allow the observation of the droplets. Leakage from the chamber is prevented by the use of O-rings seals on the windows and inserts. A nozzle is printed in the photoresin IP-L with a sub-micrometric resolution using a 3D-printer Photonic Professional GT from Nanoscribe company and then glued onto the tip of the injection glass capillary. Capillaries (Postnova Analytics) are coated with a polyimide film on the external diameter, ensuring a very high mechanical resistance. They are held into the inserts in such a way that for all combinations, alignment is guaranteed and a fixed gap between the nozzle and the extraction capillary is maintained. As demonstrated later, one can then change in a few minutes the couple of nozzle and extraction capillary in order to have access to other ranges of droplet radii, which represents a great advantage as compared to most of other glass capillary devices. Connections to the fluids inlets and outlets are ensured by standard Upchurch fittings directly screwed on the inserts for the connection to the capillaries and on the chamber for the continuous phase supply and bleed.

As shown in Fig. 2d, the input capillary supporting the nozzle provides the dispersed phase while the output capillary collects droplets carried by the continuous phase. At the entrance of the output capillary, the continuous phase pressurised in the chamber encounters a dramatic acceleration due to the change of section and therefore squeezes the dispersed phase flowing out of the nozzle, resulting in the formation of droplets (see Fig. 2e).

Fluids are injected in the device using a pressure controller (MFCS-EZ, Fluigent) and the flow rates are measured using flow-meters (Flow Unit, Fluigent), as shown in Fig. 2f. A high-speed camera (MotionPro Y3, IDT) operating up to 10000 frames per second is connected to a microscope with a 10x magnification to visualise the droplets. Recorded images are then processed with a Python script to detect the contour of the droplets and determine their size.

## Experiments

To demonstrate the operation of the device, we used two nozzles of tip radii $R_n = 15$ or $45~\upmu$m, coupled with an extraction capillary of internal radius $R_e= 75$ or $225~\upmu$m, respectively. The distance *H* between the nozzle tip and the extraction capillary is 50 or 75 $\upmu$m, respectively, the thickness *e* of the walls of the extraction capillary is 75 $\upmu$m and the external angle of the nozzle $\alpha$ taken perpendicularly from the main flow axis is 50$^\circ$. We used ultrapure water, obtained from a Sartorius water filtration system, as dispersed phase and light mineral oil, purchased from Sigma-Aldrich, as continuous phase. Both phases are filtered with PTFE 0.45 $\upmu$m syringe filters. The interfacial tension of the bare interface between the two fluids was measured to be 49.5 mN/m using the pendant drop method (tensiometer Kruss DSA-100). Capillary-based devices do not necessitate the use of a surfactant to generate droplets, in both water in oil (W/O) and oil in water (O/W) cases. Even though surfactants can be used to stabilise the emulsion after the droplets are collected and stored, the literature mentions that its presence does not affect the droplet formation (see for instance Erb *et al.*^46^). Indeed, adsorption times at the interface of the liquids are on a typical scale of tens of milliseconds whereas the droplet formation occurs in a few milliseconds. To validate this assumption in the case of our device, droplets generated with and without surfactant (Span 80, 2% w/w in the continuous phase) have been compared, with no difference. On the contrary, surfactants however do have an influence on the droplet formation in planar configuration, as they modify the contact angle at the triple line between the continuous phase, the disperse phase and the wall. This triple line could be of major nuisance in droplet formation. Fluid properties and geometrical parameters for two different couples of nozzle and extraction capillary mentioned above are listed in Table 1. Subscripts *d* and *c* refer to the dispersed and continuous phases, respectively, $\mu$ being the dynamic viscosity, $\gamma$ the interfacial tension and $\rho$ the density of the fluids.

**Table 1:** Fluid properties and geometrical parameters used in this work with water and oil for the dispersed and the continuous phases, respectively. “Couple” refers to a specific combination of a nozzle and an extraction capillary.

Fluid properties		Geometrical parameters		
			Couple 1	Couple 2
$\mu _d$ (mPa.s)	1	$R_n$ ($\upmu$m)	15	45
$\mu _c$ (mPa.s)	23	$R_e$ ($\upmu$m)	75	225
$\gamma$ (mN/m)	49.5	*H* ($\upmu$m)	50	75
$\rho _d$ (kg/m$^3$)	1000	*e* ($\upmu$m)	75	
$\rho _c$ (kg/m$^3$)	860	$\alpha$ (deg)	50	

**Figure 3:**
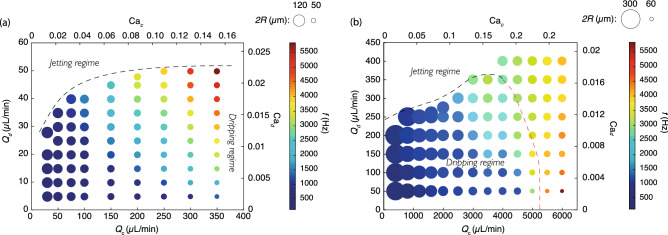
Size and production frequency of water droplets in mineral oil as a function of the continuous and dispersed flow rates $Q_c$ and $Q_d$, respectively, for the parameters given in Table 1, with (**a**) couple 1 and (**b**) couple 2 for the geometries. The values of the capillary numbers for the dispersed and continuous phases, as defined in Eq. (9) and with ${\mathrm{Ca}}_d={\bar{Q}}\, {\mathrm{Ca}}_c$, are also given on the right and top axes, respectively. The dashed lines separate the dripping to jetting regimes. In the case the jetting regime is reached for high $Q_c$ in the region on the right of the red dashed line in (**b**), it still makes sense to plot the data because of a weak polydispersity characterized by a coefficient of variation (CV) around 4%.

Considering the first couple of nozzle and extraction capillary (see dimensions in Table 1), for each value of the oil flow rate $Q_c$ between 20 and 350 $\upmu$L/min, the flow rate of the dispersed water $Q_d$ is increased until it reaches the dripping-jetting transition. The results are shown in Fig. 3a where the size of the bullet is proportional to the size of the droplet and the color scale indicates the frequency of droplets generation.We observe with the geometry of couple 1 that droplets are generated in a range of radius *R* from 25 to 60 $\upmu$m with a high monodispersity (coefficient of variation: CV $< 1$%). For a given $Q_d$, the droplet radius decreases by increasing $Q_c$, while for a given $Q_c$, it remains very stable as $Q_d$ is increased, with a maximum radius variation of 15% at $Q_c = 350 ~\upmu$L/min. Additionally, as the system operates for $Q_c \gtrsim 200$ $\upmu$L/min, the dripping-jetting transition (dashed line in Fig. 3a) reaches a plateau indicating that the dripping-jetting transition is only determined by the geometry in this region. Yet the droplet generation frequency increases to reach a maximum of 5500 Hz in this case. We have also tested the sensitivity of the device to small geometrical modifications such as a small misalignment of both capillaries, namely of about a fraction of the nozzle tip radius, i.e. $<R_n/4$. The influence of the distance *H* between the nozzle tip and the extraction capillary has also been tested in the range 0 to 2.5 $R_n$. In both tests, droplet radii have been found comparable to these obtained with the nominal values of the parameters.

**Figure 4:**
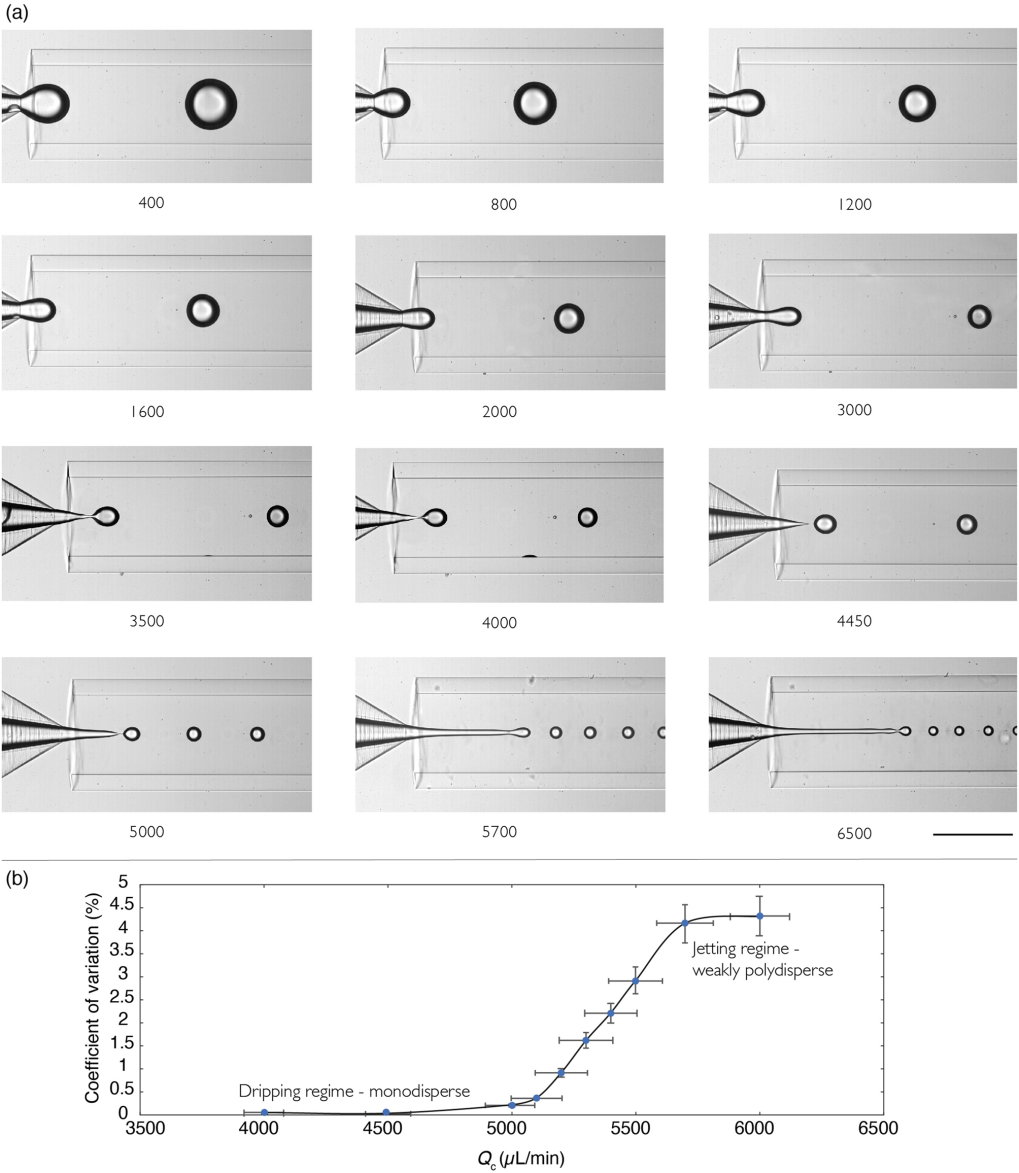
Water droplets in mineral oil using couple 2. (**a**) $Q_d$ is fixed at 50 $\upmu$L/min while the values of $Q_c$ are reported below each image. The scale bar is 450 $\upmu$m. Capillary numbers as defined in Eq. (9) and with ${\mathrm{Ca}}_d={\bar{Q}} {\mathrm{Ca}}_c$, are ${\mathrm{Ca}}_d=0.0024$ and ${\mathrm{Ca}}_c=4.9\times 10^{-5}Q_c$, with $Q_c$ in $\upmu$L/min. (**b**) Coefficient of variation (CV) versus $Q_c$ for $Q_d$ fixed at 50 $\upmu$L/min. The transition between the dripping monodisperse regime and the weakly polydisperse jetting regime occurs for $Q_c \approx 5200\pm 500$ $\upmu$L/min.

With the geometry of couple 2 (see dimensions in Table 1), droplets can be generated in a range of radius from 60 to 300 $\upmu$m (see Fig. 3b). While only the dripping regime could be observed for couple 1, because of the limitation of the maximum pressure drop attainable, higher flow rates of the continuous phase $Q_c$ could be reached with couple 2 corresponding to a less resistive geometry. Consequently, two dripping-jetting transitions could be observed : as for couple 1, a dripping-jetting transition at increasing $Q_d$ (black dashed line in Fig. 3b) and another dripping-jetting transition when $Q_c$ is increased above a threshold value (red dashed line in Fig. 3b). As shown in Fig. 4, this second transition at high $Q_c$ separates a monodisperse regime with CV $< 1$% from a polydisperse regime with CV $> 4$%, a value still much smaller than typical CV values observed in the jetting regime at sufficiently large $Q_d$, usually larger than 10%. Indeed, in this jetting mode, the jet is uniform in radius and acts like a noise amplifier, therefore being more sensitive to external noise. But in the jetting regime at sufficiently large $Q_c$, the jet is non-uniform as it is stretched in the flow direction, which has been shown by Gordillo *et al.*^47^ to have a stabilizing effect on the Rayleigh-Plateau instability. This plays in favor of a mode selection at the tip of the jet, resulting in a lower polydispersity visible on the images of Fig. 4a where no polydisperse regime seems to be reached whatever the value of $Q_c$, reminiscent to tip streaming regime^25^. Actually, a statistical analysis of the diameter of a large number of droplets ($>200$) allows to highlight the steep transition in CV that occurs for $Q_c$ at around $5200\pm 500$ $\upmu$L/min and plotted in Fig. 4b. This transition is in good agreement with the prediction of the numerical model described below.

## Modelling

With the goal to predict the droplet radius generated in the dripping regime using the Raydrop, we propose in this section to model the non-embedded co-flow-focusing configuration in transient and then using the quasi-static approach. After a validation with the experimental results, we show how to determine the droplet size in the dripping regime, as well as the dripping to jetting transition, when varying the continuous flow rate alone.

### Transient

**Figure 5:**
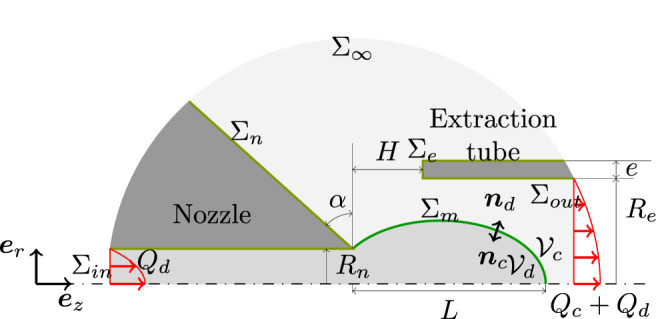
Sketch of the droplet generation inside a Raydrop.

Geometry of the non-embedded co-flow-focusing configuration is considered in an axisymmetric coordinate system (*r*, *z*), with *r* and *z* the radial and axial coordinates, respectively, as sketched in Fig. 5.The properties and parameters for the two phases will be given with the generic subscript *i* referring to *d* and *c* for the dispersed and the continuous phases, respectively. Each phase has thus a density $\rho _i$, a dynamic viscosity $\mu _i$ and a flow rate $Q_i$. The time-dependent volumes of the two phases are denoted by $\mathscr {V}_i$ and are separated by the surface $\Sigma _m$, characterized by an interfacial tension $\gamma$. The surface of the nozzle and the extraction capillary are denoted by $\Sigma _n$ and $\Sigma _e$, respectively. The domain considered is truncated at $\Sigma _{\infty }$, $\Sigma _{\mathrm{{in}}}$ and $\Sigma _{\mathrm{{out}}}$, which are sufficiently far from the droplet generation zone to not affect the results. The dispersed flow rate $Q_d$ is injected in the form of a fully developed Poiseuille flow through the capillary of the nozzle at $\Sigma _{\mathrm{{in}}}$, and both fluids leave the device through the cross-section of the extraction capillary $\Sigma _{\mathrm{{out}}}$ at a flow rate $Q_c+Q_d$. In addition to the geometrical parameters already defined in Table 1, one can define $L$ as the distance taken on the symmetry axis from the nozzle tip to the tip of the meniscus, as sketched in Fig. 5.

The motion of the fluids is governed by the continuity and the Navier–Stokes equations,

1 $$\begin{aligned} {\mathbf {\nabla }}\cdot {{{\mathbf{v}}}}_i = 0 \,, \quad \rho _i (\partial _t {\mathbf{v}}_i + ({{\mathbf{v}}}_i \cdot \mathbf {\nabla }) {{\mathbf{v}}}_i) = \mathbf {\nabla }\cdot \mathbf {\tau }_i \,, \quad \text{ at } \mathscr {V}_i \,, \end{aligned}$$

where ${{\mathbf{v}}}_i$ is the velocity vector and $\mathbf {\tau }_i=-p_i {\mathscr {I}}+ \mu _i \left[ \mathbf {\nabla }{{\mathbf{v}}}_i + \left( \mathbf {\nabla }{{\mathbf{v}}}_i \right) ^T \right]$ is the stress tensor of the phase *i* with $p_i$ being the pressure field and ${\mathscr {I}}$ the identity matrix. Defining ${\mathbf{n}}_i$ as the outer normal to the domain $\mathscr {V}_i$ at any of its boundaries, as sketched in Fig. 5, the no-stress boundary condition at $\Sigma _{\infty }$ writes

2 $$\begin{aligned} {\mathbf{n}}_c \cdot \mathbf {\tau }_c = \mathbf {0}\qquad \text{ at } \Sigma _{\infty } \,. \end{aligned}$$

The flow being considered as fully developed (i.e. parallel flow), upstream of the nozzle as well as downstream of the extraction capillary, we have

3a $$\begin{aligned} {\mathbf{n}}_d \cdot \mathbf {\tau }_d&= P_d \,{\mathbf{n}}_d \qquad \text{ at } \Sigma _{\mathrm{{in}}} \,, \end{aligned}$$

3b $$\begin{aligned} {\mathbf{n}}_c \cdot \mathbf {\tau }_c&= P_c \, {\mathbf{n}}_c \, \qquad \text{ at } \Sigma _{\mathrm{{out}}} \,, \end{aligned}$$

where the pressures, $P_d$ and $P_c$, correspond to the ones imposing the given flow rates

4a $$\begin{aligned}&\int _{\Sigma _{\mathrm{{out}}}} {\mathbf{e}}_z \cdot {{\mathbf{v}}}_c \, \mathrm{{d}}\Sigma = Q_d+ Q_c \,, \end{aligned}$$

4b $$\begin{aligned}&{\mathscr {V}}_d(t) - {\mathscr {V}}_d(0) = Q_d \, t, \end{aligned}$$

with *t* the time. No-slip boundary conditions at the nozzle and at the extraction capillary are applied

5a $$\begin{aligned} {{\mathbf{v}}}_c&= 0 \qquad \text{ at } \Sigma _{n} \cup \Sigma _{e} \,, \end{aligned}$$

5b $$\begin{aligned} {{\mathbf{v}}}_d&= 0 \qquad \text{ at } \Sigma _{n} \,. \end{aligned}$$

The continuity of velocity and stress balance at the interface, which is pinned at the nozzle tip, writes

6a $$\begin{aligned} {{\mathbf{v}}}_d&= {{\mathbf{v}}}_c \,&\qquad&\text{ at } \Sigma _{m} \,, \end{aligned}$$

6b $$\begin{aligned}&{\mathbf{n}}_d \cdot \mathbf {\tau }_d + {\mathbf{n}}_c \cdot \mathbf {\tau }_c = \mathbf {\nabla }_S \cdot \left( \gamma {\mathscr {I}}_S \right) \,&\qquad&\text{ at } \Sigma _{m} \,, \end{aligned}$$

where ${\mathscr {I}}_S= {\mathscr {I}}- {\mathbf{n}}_i {\mathbf{n}}_i$ is the surface identity tensor. The kinematic condition writes

7 $$\begin{aligned} {{\mathbf{v}}}_d \cdot {\mathbf{n}}_d&= \partial _t \mathbf {x}\cdot {\mathbf{n}}_d \, \qquad \text{ at } \Sigma _{m} \,, \end{aligned}$$

where $\mathbf {x}\in \Sigma _{m}$ and $\partial _t \mathbf {x}\cdot {\mathbf{n}}_d$ represents the normal velocity of the interface.

The instantaneous equivalent radius of the meniscus $R_{m}(t)$ is defined as the radius of a sphere of a volume equivalent to the one of the dispersed phase beyond the nozzle and bounded by the meniscus surface $\Sigma _m$, namely

8 $$\begin{aligned} \frac{4}{3} \pi R_{m}^3 (t) = \frac{1}{2} \int _{\Sigma _m(t)} r \,\mathbf {e}_r \cdot {\mathbf{n}}_d \, \mathrm{{d}}\Sigma \,. \end{aligned}$$

**Figure 6:**
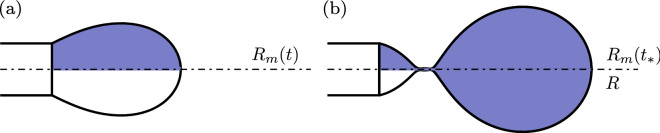
Sketch of the instantaneous equivalent radius of the meniscus $R_{m}(t)$, and of the drop *R*, (**a**) for an arbitrary time *t* and (**b**) for the pinch-off time $t_*$.

It should be noted that at the pinch-off time $t_*$, the volume preceding the neck is the one corresponding to the droplet $\frac{4}{3} \pi R^3$. In Fig. 6, the volumes of revolution reconstructed from the shaded areas are related to $R_{m}$ and *R*. The meniscus shape and volume for an arbitrary time and for the pinch-off time are sketched in Fig. 6a,b, respectively. The instantaneous equivalent radius of the meniscus $R_{m}(t)$ is time-dependent and shadowed at both times, whereas the radius of the drop *R* is only defined at the pinch-off time $t_*$, and is therefore not time-dependent.

The equations are non-dimensionalised using $R_n$ for the length scale, $\gamma / \mu _c$ for the velocity scale and $\gamma /R_n$ for the pressure scale, leading to the following dimensionless flow parameters

9 $$\begin{aligned} \displaystyle {\mathrm{Ca}}_c = \frac{\mu _c Q_c}{\gamma \pi R_{e}^2}\,, \quad {\bar{Q}}= \frac{Q_d}{Q_c}\,,\quad {\mathrm{Ca}}_d \equiv {\bar{Q}} \, {\mathrm{Ca}}_c=\frac{\mu _c Q_d}{\gamma \pi R_{e}^2} \,, \quad {\bar{R}}_e= \frac{R_e}{R_n}\,, \quad \displaystyle \lambda = \frac{\mu _d}{\mu _c}\,, \quad \mathrm{{La}}= \frac{\rho _c \gamma R_n}{\mu _c^2}\,, \quad \phi = \frac{\rho _d}{\rho _c} \end{aligned}$$

with ${\mathrm{Ca}}_c$ the capillary number for the continuous phase, $\bar{R}_e$ the radius ratio, ${\bar{Q}}$ the flow rate ratio, $\lambda$ the viscosity ratio, $\mathrm{{La}}$ the Laplace number and $\phi$ the density ratio. Finally, the other geometrical parameters are all made dimensionless relative to the diameter of the nozzle tip:

10 $$\begin{aligned} {\bar{H}} = \frac{H}{R_n}\,, \quad {\bar{e}}=\frac{e}{R_n}\,, \quad \bar{R}_{m}= \frac{R_{m}}{R_n}\,, \quad {\bar{L}} = \frac{L}{R_n} \,. \end{aligned}$$

The system of Eqs. (1)–(7) governs the droplet formation using the Raydrop. The domain variables are $p_i$ and ${{\mathbf{v}}}_i$, the surface variable is $\mathbf {x}$, and the global variables are $P_i$. This system of equations is solved using the Finite Element Method (FEM) with the help of the software Comsol and quadratic Lagrangian elements, with exception of the pressure for which linear elements have been used. For the deformable domain, the *moving mesh* application mode has been used. It implements the Arbitrary Lagrangian-Eulerian (ALE) method combined with the Boundary Arbitrary Lagrangian-Eulerian (BALE) method previously developed in Ref.^44^. In every simulation, the initial shape of the meniscus is half a sphere out of the nozzle. Transient simulations have been carried out using a first-order backward-Euler time discretization until the pinch-off time. The criteria for the pinch-off is when the neck radius has reached 2% of the nozzle tip radius.

Finally, we note that most of the results presented below have been obtained with the Stokes equations, i.e. with the left-hand side of the momentum balance in Eq. (1) set to $\mathbf{0}$. We have indeed checked numerically (not shown) that for the maximum Reynolds number corresponding to our experimental conditions (see Table 1), or equivalently for a maximum Laplace number $\mathrm{{La}}$ of about 100, inertia has no influence on these results. Therefore, all results presented below are for $\mathrm{{La}}=0$, unless specified otherwise.

### Quasi-static approach

In general, the system behavior is transient but considering the quasi-static (QS) limit is of great interest. This limit corresponds to the situation of $Q_d \rightarrow 0$, meaning that the flow of the dispersed phase is negligible, and thus set to zero, i.e. $Q_d=0$. Consequently, the system of Eqs. (1)–(7) can be solved for stationary solutions, cancelling the time-derivatives in Eqs. (1) and (7), and parametrizing the volume of the liquid meniscus with its quasi-static equivalent radius $\bar{R}_{m}$, instead of using time *t* in Eq. (4b). This QS approach thus facilitates the parametrical analysis and allows to provide an explanation to the underlying mechanism behind the drop formation. By convenience, quasi-static simulations have been carried out using parametric continuation in $\bar{L}$ instead of ${\mathrm{Ca}}_c$, this latter being considered as a simulation output. As for the transient simulation, the initial shape for the start of the continuation procedure is assumed to be half a sphere. But afterwards, at each step of the procedure the initial shape is taken by continuation on the parameter *L*¯  from the previous solution. Hence the quasi-static solution does not depend on the initial guess for the droplet shape.

For comparison purposes, we show in Table 2 the correspondence between the equivalent radii obtained from the transient and the quasi-static approaches.

**Table 2:** Dimensionless equivalent radii obtained in transient, as defined in Fig. 6, and with the quasi-static approach.

**Transient (Ca**_***d***_** > 0)**	
Equivalent radius of the meniscus	$\bar{R}_{m}(t)$
$\quad ...$ at pinch-off time $t_*$	$\bar{R}_{m}(t_*)$
$\quad \quad ...$ preceding the pinch-off location	$\bar{R}$
$\qquad \quad ...$ in the QS limit for ${\mathrm{Ca}}_d \rightarrow 0$	$\bar{R}^{\mathrm{QS}}$
**Quasi-static (Ca**_**d**_ **= 0)**	
Equivalent radius of the meniscus	$\bar{R}_{m}$
$\quad ...$ at the folding bifurcation point	$\bar{R}_{m}^D$

### Model validation

**Figure 7:**
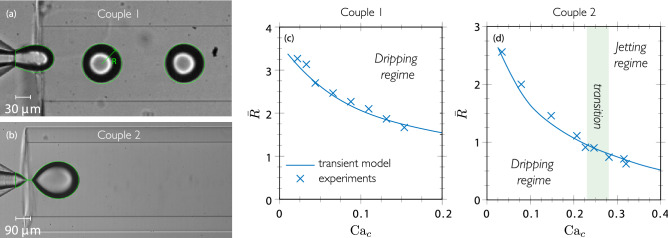
Comparison between transient simulations (green lines) and experimental pictures for the formation of water drops in mineral oil with properties reported in Table 1: (**a**) meniscus for $Q_c=150\,\mathrm {\mu L/min}$ (${\mathrm{Ca}}_c=0.066$) and $Q_d=25\,\mathrm {\mu L/min}$ (${\mathrm{Ca}}_d=0.01$); (**b**) meniscus at pinch-off for $Q_c=700\,\mathrm {\mu L/min}$ (${\mathrm{Ca}}_c=0.034$) and $Q_d=20\,\mathrm {\mu L/min}$ (${\mathrm{Ca}}_d=0.001$); (**c**,**d**) comparisons of droplet size for various values of ${\mathrm{Ca}}_c$ in the dripping regime for (**c**) and showing for (**d**) the experimental dripping-jetting transition (shaded area), as identified in Fig. 4.

The model in the Stokes limit computed first in transient is validated by comparison with experimental data, as shown in Fig. 7. Figure 7a displays the superposition of the droplet shape with a circle of radius *R* (green line) obtained from the equivalent radius of the meniscus $R_m$ predicted by the model for couple 1. In Fig. 7b, the interfacial shape of a drop in formation is shown just before the pinch-off of the meniscus for couple 2. In both cases, an excellent agreement is observed. In Fig. 7c,d, the experimental and numerical drop sizes are compared for a wide range of ${\mathrm{Ca}}_c$, showing again an excellent agreement for the two geometries (couples 1 and 2). It can be observed that as the continuous flow rate increases, the generated drop becomes smaller. The shaded area in Fig. 7d corresponds to the dripping-jetting transition identified experimentally in Fig. 4 in the range $0.23 \lesssim {\mathrm{Ca}}_d \lesssim 0.28$. Finally, note that $Q_d$ has been kept small enough to ensure ${\bar{Q}} \ll 1$, such as it should have no influence on the droplet size, then suggesting that the QS limit is applicable.

**Figure 8:**
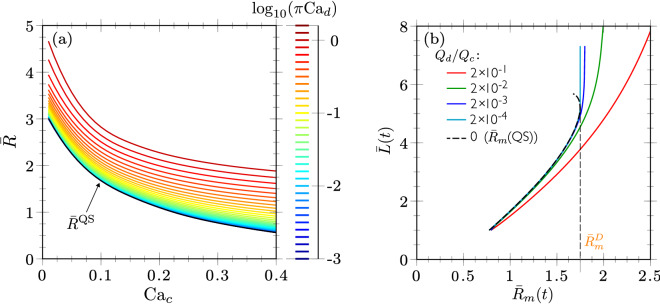
(**a**) Influence of the ${\mathrm{Ca}}_d$ on the generated drop radius as a function of ${\mathrm{Ca}}_c$ computed in transient considering couple 1 (see Table 1); ${\bar{R}}^{\mathrm{QS}}$ in black line corresponds to ${\mathrm{Ca}}_d = 4\times 10^{-6}\pi ^{-1}$, i.e. the quasi-static limit. (**b**) Transient evolution of the meniscus length $\bar{L}$ as a function of the meniscus equivalent radius $\bar{R}_{m}$ for different values of ${\bar{Q}}=Q_d/Q_c$ and ${\mathrm{Ca}}_c=0.1$, considering couple 1 (see Table 1). The equivalent radius of the meniscus calculated with quasi-static simulations is plotted in black dashed line.

In order to verify the range of validity of the QS limit, Fig. 8a shows the predictions of the droplet size in transient for various values of ${\mathrm{Ca}}_d$, as well as in the QS limit as represented by the black solid line, denoted as $\bar{R}^{\mathrm{QS}}$ and effectively obtained for ${\mathrm{Ca}}_d = 4\times 10^{-6}\pi ^{-1}$.This limit lies slightly below the numerical curve plotted in Fig. 7c, as represented by the orange area in Fig. 8a for $\log _{10}(\pi {\mathrm{Ca}}_d) \approx -0.67$. Figure 8a shows that for increasing values of ${\mathrm{Ca}}_d$, the system leads to a monotonous (yet logarithmic) increase of the drop size, as compared to the QS limit prediction.

In order to understand how the flow rate of the continuous phase affects the size of the drop as a function of the geometry and of the properties of the given pair of fluids, the comparison between the transient and the quasi-static solutions is further investigated. In Fig. 8b, the dynamics of the meniscus shape predicted from a quasi-static simulation (${\bar{Q}} = 0$), as well as from transient simulations of several values of ${\bar{Q}}$ is presented for a given value of ${\mathrm{Ca}}_c$.

Parameters $\bar{L}$ and $\bar{R}_{m}$ were chosen as representative quantities of the meniscus shape and are plotted as trajectory lines following the time during the formation process. One notices that the lines diverge as ${\bar{Q}} \rightarrow 0$. Remarkably, solving the model using the quasi-static simulation for exactly ${\bar{Q}} = 0$ depicts the existence of a turning-point corresponding to a folding bifurcation. This is analogous to the detachment of a pendant drop as discussed in the introduction and further developed below. We note $\bar{R}_{m}^D$ the value of $\bar{R}_{m}$ at the turning-point, as labeled in Table 2.

In the transient simulations with finite values of ${\bar{Q}}$, it can be observed (i) that the meniscus is less elongated for larger $\bar{Q}$ and given $\bar{R}_{m}$, especially close to the turning-point, which naturally leads to larger droplets as shown in Fig. 8a, and (ii) that larger menisci, i.e. $\bar{R}_{m}>\bar{R}_{m}^D$, can be attained for finite dispersed flow rates. At that stage, $\bar{L}$ exhibits a large increase for a small increase of $\bar{R}_{m}$, due to the elongation concentrated in the neck region shown in Fig. 7b, i.e. the menisci evolve towards the pinch-off, leading to the formation of a drop of size $\bar{R}$.

**Figure 9:**
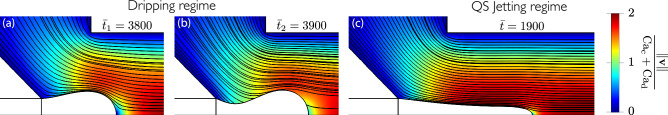
Axisymmetric simulations in transient using couple 1 (see Table 1) and ${\mathrm{Ca}}_d=8 \cdot 10^{-5}$ (QS limit). The flow field is represented by streamlines and rescaled velocity norm $||{{\mathbf{v}}}_c||/({\mathrm{Ca}}_c+{\mathrm{Ca}}_d)$ as color code. The timescale is $R_n\mu _c/\gamma =7\,\upmu$s. (**a**,**b**) Dripping regime with ${\mathrm{Ca}}_c=0.1$ for a time before (**a**) and after (**b**) the folding bifurcation; (**c**) QS-jetting regime for ${\mathrm{Ca}}_c=0.3>{\mathrm{Ca}}_c^{*}$ (see section Inviscid droplets for details).

In Fig. 9, the flow field and meniscus shape are represented at two different times. While in Fig. 9a the volume exhibits a quasi-steady meniscus since $\bar{R}_{m}(\bar{t}_1)<\bar{R}_{m}^D$, in Fig. 9b the volume is no longer in equilibrium since $\bar{R}_{m}(\bar{t}_2) > \bar{R}_{m}^D$, leading to the formation of a neck. On the one hand, it can be observed that, for a quasi-static meniscus, streamlines are tangent to the meniscus revealing the quasi-static character of the flow, i.e. the right-hand side term of Eq. (7) is negligible and thus set to zero, i.e. $Q_d=0$. On the other hand, for larger volumes, the quasi-static meniscus no longer exists and it exhibits a neck which eventually breaks for later times. In this situation, the meniscus evolves dynamically as revealed by the streamlines which are no longer tangent to the meniscus, and the right-hand side of Eq. (7) is not negligible anymore, even in the limit of ${\bar{Q}} \rightarrow 0$. Finally, the transient solution shown in Fig. 9c for a larger ${\mathrm{Ca}}_c$ depicts the shape of a jet with streamlines again parallel to the interface, thus suggesting a quasi-static solution like in Fig. 9a. As it will be shown below, this solution corresponds to the jetting regime in the quasi-static limit, occurring at a ${\mathrm{Ca}}_c$ larger than the transition value, denoted by ${\mathrm{Ca}}_c^*$.

## Parametric analysis

Results obtained so far support the quasi-static approach for two reasons: (i) theoretically, the occurrence in Fig. 8b of a folding bifurcation in this limit allows for transition tracking between dripping and jetting modes; (ii) experimentally, the size of the droplets shown in Fig. 3 barely varies with $Q_d$ at constant $Q_c$, suggesting that size predictions in the limit of $Q_d \rightarrow 0$ should also apply for finite $Q_d$, which then only influences the frequency of droplet generation, at least to some extent. In this section, quasi-static simulations are therefore used as a predictive tool to evaluate the influence of fluid properties and geometric parameters on the drop formation.

### Inviscid droplets

**Figure 10:**
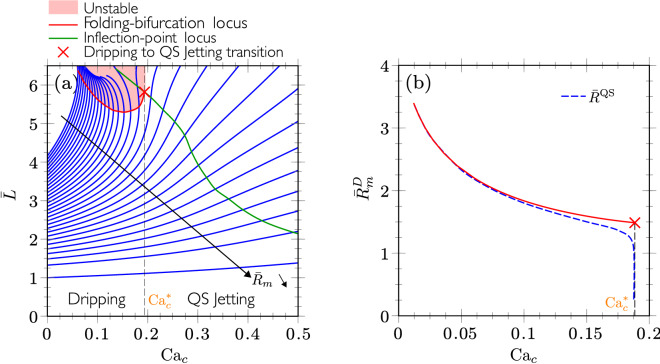
Dripping mode obtained with quasi-static simulations for the geometry of couple 1 and in the inviscid limit $\lambda =0$: influence of (**a**) $\bar{R}_{m}$ and ${\mathrm{Ca}}_c$ on $\bar{L}$ and (**b**) ${\mathrm{Ca}}_c$ on $\bar{R}_{m}^D$. The blue dashed line represents the equivalent radius $\bar{R}^{\mathrm{QS}}$ as defined in Table 2, while the red cross indicates the dripping to jetting transition at ${\mathrm{Ca}}_c^*$.

Starting with the inviscid droplet limit, i.e. $\lambda =0$, we plot in Fig. 10a the quasi-static meniscus length $\bar{L}$ as a function of ${\mathrm{Ca}}_c$ for constant equivalent radii of the meniscus $\bar{R}_{m}$ (blue lines), the arrows indicating the decrease of $\bar{R}_{m}$. It can be observed that in some range of ${\mathrm{Ca}}_c$ corresponding to large values of $\bar{R}_{m}$, the curves of constant $\bar{R}_{m}$ exhibit a turning-point. The loci of the turning-points are then depicted by the red curve, as determined by $\partial {\mathrm{Ca}}_c / \partial \bar{L}|_{\bar{R}_{m}} = 0$. The shadowed region above this curve is therefore the unstable region. Consequently, a dripping occurs at a fixed ${\mathrm{Ca}}_c$ as the volume of the meniscus is quasi-statically increased up to the red curve corresponding to $\bar{R}_{m}=\bar{R}_{m}^D$. Quasi-static solution no longer exists for higher volumes, as already shown in Fig. 8b for ${\mathrm{Ca}}_c=0.1$ and $\lambda =1/23$.

Notably, the locus of turning points stops at ${\mathrm{Ca}}_c^*$ (red cross in Fig. 10a), thus representing the maximum ${\mathrm{Ca}}_c$ for which the dripping regime in the QS limit occurs. Indeed, as a matter of fact, this maximum no longer exists on the right of the locus of the inflection-point (green curve), determined by $\partial ^2 {\mathrm{Ca}}_c / \partial \bar{L}^2 |_{\bar{R}_{m}} = 0$. Quasi-static and transient simulations show that for ${\mathrm{Ca}}_c>{\mathrm{Ca}}_c^*$, a jet is established as was shown in Fig. 9. In Fig. 10a, and for a fixed ${\mathrm{Ca}}_c > {\mathrm{Ca}}_c^*$, as the volume of the meniscus increases, the tip of the jet advances and the jet is formed behind. Remarkably, the form of the jet does not vary behind the tip, which recalls the steady shape of the fluid cone in the tip streaming process^25,48^.

In Fig. 10b, the folding bifurcation locus is shown in the ($\bar{R}_{m}^D$,${\mathrm{Ca}}_c$)-plane. It can be observed that the equivalent radius is smaller for larger values of the continuous flow rate, as the viscous forces exerted by the continuous phase on the meniscus is larger, inducing the detachment of a smaller droplet. This recalls the analogy with the pendant droplet, provided the hydrodynamic forces are substituted to the gravity forces. We also plot in blue dashed line the equivalent radius $\bar{R}^{\mathrm{QS}}$ corresponding to the volume of revolution preceding the neck, as obtained in transient in the quasi-static limit, i.e. for ${\mathrm{Ca}}_c \rightarrow 0$ (see Table 2). As ${\mathrm{Ca}}_c$ increases, the position of the neck moves from the nozzle at ${\mathrm{Ca}}_c \rightarrow 0$ to the tip of the meniscus at ${\mathrm{Ca}}_c^*$, for which the neck disappears. This explains the dramatic decrease of $\bar{R}^{\mathrm{QS}}$ when approaching the jetting transition, even though it only occurs for inviscid droplets and is therefore not really physical. Indeed, a finite viscosity of the dispersed phase is found to regularise this singularity (see Fig. 8a).

Now, for lower ${\mathrm{Ca}}_c$, $\bar{R}^{\mathrm{QS}}$ is shown to follow the same behavior than $\bar{R}_{m}^D$, even though it remains smaller because of the volume that remains attached to the nozzle behind the neck. Consequently, it confirms that except near the dripping-jetting transition, the value $\bar{R}_{m}^D$ is well representative of the drop radius determined in transient in the quasi-static limit, but without relying on the pinch-off formation.

Finally, it is worth mentioning that the equivalent radius of the meniscus for dripping in Fig. 10b can be correlated by

11 $$\begin{aligned} \bar{R}_{m}^D \approx 0.84 \,{\mathrm{Ca}}_c^{-0.34}\qquad (\lambda =0). \end{aligned}$$

The prefactor and mostly the exponent are similar to those found by Lan *et al.*^31^ for dripping in a co-flow configuration, namely $\bar{R}=1.07 \, {\mathrm{Ca}}^{-0.34}Re_d^{0.06}$ with $Re_d=2\rho _dQ_d/(\pi \mu _d R_n)$ the Reynolds number of the dispersed flow. Apart from the slight influence of inertia, i.e. $Re_d^{0.06}\approx 1.2$, not included in Eq. (11) since obtained for a Stokes flow and $\lambda =0$, it shows that the drop size predictions in the dripping mode are comparable in co-flow and co-flow-focusing.

### Viscous droplets

**Figure 11:**
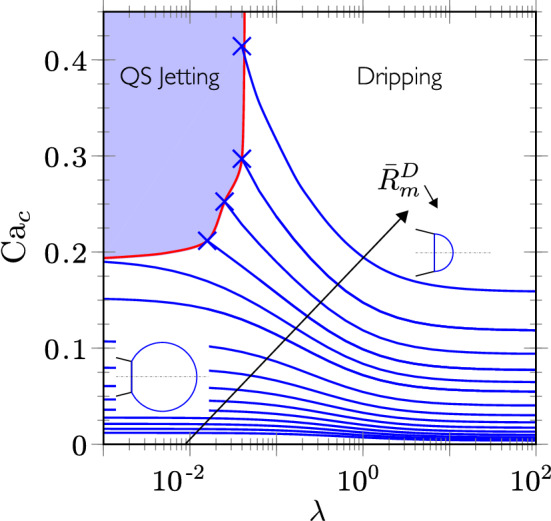
Influence of $\lambda$ in the quasi-static dripping regime on the value of ${\mathrm{Ca}}_c$ necessary to produce a drop from a meniscus equivalent radius $\bar{R}_{m}^D$ (blue lines). The red curve corresponds to the dripping-jetting transition for ${\mathrm{Ca}}_c^*$. The geometry of couple 1 has been used. The two sketches show the equivalent spherical cap for $Ca_c=0$ for large and small ${\bar{L}}$, i.e. large and small volumes.

Next, the influence of the viscosity ratio $\lambda$ on the meniscus equivalent radius is considered. Each blue line in Fig. 11 corresponds to a constant meniscus equivalent radius $\bar{R}_{m}^D$ in the ($\lambda$,${\mathrm{Ca}}_c$)-plane, while the black arrow indicates the decrease of the meniscus volume. We observe that, as $\lambda$ increases, smaller values of ${\mathrm{Ca}}_c$ are needed to generate a drop of the same size. This can be explained by the fact that for inviscid droplet, only the normal component of the viscous stresses exerted at the interface contributes to the net force responsible for the droplet detachment, while, as $\lambda$ increases, the tangential (or shear) component is added to the force, provoking the detachment of smaller droplets. For the same reason, the dripping-jetting transition occurs for larger values of ${\mathrm{Ca}}_c^*$ as $\lambda$ increases, as shown by the red curve in Fig. 11, which starts from the value of ${\mathrm{Ca}}_c^*=0.188$ in the limit of inviscid droplets, to large and possibly asymptotically infinite value for viscous droplets corresponding to $\lambda \gtrsim 5\times 10^{-2}$. It thus appears that beyond this value, the viscous shear stress exerted on the droplet is always large enough to ensure the dripping mode.

Practically, the influence of $\lambda$ on ${\mathrm{Ca}}_c$ for the dripping mode can be described by a correlation involving the limits for $\lambda \rightarrow 0$ and $\lambda \rightarrow \infty$,

12 $$\begin{aligned} {\mathrm{Ca}}_c (R_m^D,\lambda )=\frac{\lambda _* (\bar{R}_{m}^D) \,{\mathrm{Ca}}_{c,0} (\bar{R}_{m}^D) + \lambda \,{\mathrm{Ca}}_{c,\infty } (\bar{R}_{m}^D) }{\lambda _* (\bar{R}_{m}^D) + \lambda } \,, \end{aligned}$$

where ${\mathrm{Ca}}_{c,0}$ and ${\mathrm{Ca}}_{c,\infty }$ are the ${\mathrm{Ca}}_c$ numbers that produce menisci of size $\bar{R}_{m}^D$ in both limits, respectively, whereas the transition is centered at $\lambda _*$. These functions have been obtained numerically (see Supplementary Information), the correlations of which are ${\mathrm{Ca}}_{c,0}\approx 0.5952 (\bar{R}_{m}^D)^{-2.936}$ (as inverted from Eq. (11)), ${\mathrm{Ca}}_{c,\infty }\approx 0.1666 (\bar{R}_{m}^D)^{-2.496}$ and $\lambda _* \approx 0.09497(\bar{R}_{m}^D)^{1.609}$, applicable in the range $1.5 \lesssim \bar{R}_{m}^D \lesssim 3.5$. Using these correlations in Eq. (12) allows to estimate the dimensionless droplet size $\bar{R}_{m}^D$ for a given viscosity ratio $\lambda$ and a given capillary number of the continuous phase ${\mathrm{Ca}}_c$, provided the geometry verifies the dimensionless parameters corresponding to couple 1, namely ${\bar{R}}_e=5$, ${\bar{H}}=3.33$, ${\bar{e}}=5$ and $\alpha = 50^\circ$. The sensitivity of the droplet size on these parameters is analysed in the next section.

Figure 11 also shows that droplets generated in the highly viscous limit ($\lambda =10^2$) are always smaller than those generated in the almost inviscid limit ($\lambda =10^{-3}$), for a fixed ${\mathrm{Ca}}_c$, because of the additional shear stresses contributing to the force involved in the dripping mechanism. This argument should also be completed by the fact that if the viscosity of the dispersed phase increases, the flow resistance at the neck of the droplet increases, reducing the flow towards the droplet, hence contributing to produce smaller droplet size too^31^.

### Influence of geometrical parameters

The quasi-static approach is now used to study the influence of the geometrical parameters $\bar{R}_{e}$, $\bar{H}$ and $\alpha$ (see Fig. 5) as compared to the nominal values corresponding to the geometry of couple 1 (see Table 1), with $\lambda =1/23$.

**Figure 12:**
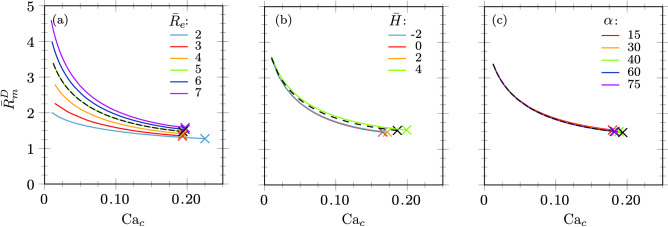
Influence of the geometrical parameters on the droplet size for $\lambda =1/23$: (**a**) the *unconfinement parameter* $\bar{R}_{e}$, (**b**) the *non-embedment parameter* $\bar{H}$ and (**c**) the nozzle inclination $\alpha$ are varied around the nominal values of couple 1 represented in dashed black line for ${\bar{R}}_e = 5$, ${\bar{H}} = 3.33$ and $\alpha = 50^\circ$.

It can be observed in Fig. 12a that a smaller ratio of the extraction capillary diameter with the nozzle diameter leads to smaller drops for the same ${\mathrm{Ca}}_c$, since the viscous stresses exerted by the continuous phase on the meniscus obviously increases with the confinement, i.e. as ${\bar{R}}_e$ decreases. Accordingly, we name ${\bar{R}}_e$ the *unconfinement parameter*. On the contrary, the dripping-jetting transition at ${\mathrm{Ca}}_c^*$ (crosses) is almost insensitive to the radius of the extraction capillary, except for ${\bar{R}}_e < 3$, indicating that strong confinement can displace the dripping to jetting transition towards larger value of $Ca_c$ and eventually enhance the dripping regime.

Figure 12b shows that the meniscus equivalent radius is quite insensitive to $\bar{H}$ for $\bar{H}<2$, above which a larger value of $\bar{H}$ leads to a larger meniscus. It can be explained by the flow field coming from an infinite medium inside a tube in absence of the dispersed phase and the nozzle, where the velocity is uniform inside the extraction capillary but decays in the axis of symmetry as moving away from the entrance. The length scale of this effect is of the order of the radius of the capillary. For larger $\bar{H}$, the flow rate should be higher to keep the same stresses around the meniscus, and thus the same droplet size. Interestingly, the dripping-jetting transition (crosses) occurs for larger ${\mathrm{Ca}}_c^*$ as ${\bar{H}}$ is increased. Reversely, this transition becomes independent on ${\bar{H}}$ for ${\bar{H}} \le 0$ (see Coefficients of Variation (CV) in Supplementary Information), i.e. when the injection capillary is embedded into the extraction capillary, as for a simple co-flow configuration. Accordingly, we name $\bar{H}$ the *non-embedment parameter*. Now, increasing ${\bar{H}}$ toward positive values seems to displace the dripping to jetting transition towards larger value of $Ca_c$, thus enhancing the dripping regime. However, we found experimentally that the dripping regime is not stable for ${\bar{H}} >4$ and rather gives place to tip-multi-breaking regime, as reported by Zhu *et al.*^49^ (see Supplementary Information).

Finally, no influence of $\alpha$ is found for the meniscus equivalent radius but small and non-monotonic influence on ${\mathrm{Ca}}_c^*$ is revealed, with a maximum around $50^\circ$, as shown in Fig. 12c. Note also that no significant influence of the thickness of the extraction tube wall, ${\bar{e}}$, could be identified, such as this parameter has been disregarded from our parametric analysis. Contrarily, we show in Supplementary Information that opening the inner diameter of the extraction capillary with a given angle can displace the quasi-static dripping to jetting transition to larger values of $Ca_c$, as already reported in planar flow-focusing^50^, thus also enhancing the dripping regime.

### Influence of inertia

**Figure 13:**
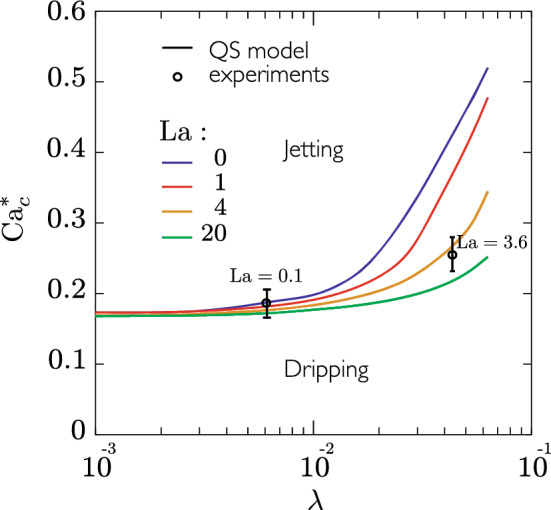
Influence of $\lambda$ on the quasi-static dripping-jetting transition at ${\mathrm{Ca}}_c^*$ for different values of the Laplace number $\mathrm{{La}}$ gauging the role of inertia. The geometry of couple 2 has been used.

As mentioned in section Modelling, all results for the drop size have been obtained with the Stokes equation, i.e. with $\mathrm{{La}}=0$, since no influence of inertia were found for $\mathrm{{La}}$ up to 100 on the drop size predictions. Nevertheless, we found that inertia can still significantly influence the occurrence of the quasi-static dripping-jetting transition. Figure 13 shows that inertia effects enhance the dripping-jetting transition, i.e. ${\mathrm{Ca}}_c^*$ decreases with increasing $\mathrm{{La}}$ for a fixed $\lambda$, and that the enhancement is more pronounced as $\lambda$ increases. On the contrary, inertia plays almost no role in the inviscid limit ($\lambda \rightarrow 0$).

Experimentally, this transition could only be identified with the geometry of couple 2 and the fluid properties given in Table 1. The corresponding point is shown in Fig. 13 for $\lambda =1/23\approx 4\times 10^{-2}$ and $\mathrm{{La}}=3.6$, with the error bar covering the range in ${\mathrm{Ca}}_c$ over which the transition was identified (see Fig. 4). Despite the large error bar, the agreement with the quasi-static approach including inertia (see orange line for $\mathrm{{La}}=4$ in Fig. 13) is satisfactory and confirms the important role of inertia for viscous droplets. Indeed, for the case of less viscous droplets, another pair of fluids corresponding to $\lambda \approx 6\times 10^{-3}$ and $\mathrm{{La}}=0.1$ has also been tested experimentally, showing again an excellent agreement with the modelling, and demonstrating a much lower influence of inertia effects for low viscosity ratios.

## Discussions and conclusions

In this paper, we have presented the Raydrop device allowing for the generation of droplets in an axisymmetric flow-focusing, that we have renamed non-embedded co-flow-focusing. This configuration, together with the quasi-static approach, has enabled a systematic parametric analysis, considering the influence of all relevant parameters. In the quasi-static dripping regime, droplet size has been shown (i) to decrease with increasing the flow rate of the continuous phase, (ii) to decrease with increasing the viscosity ratio between the dispersed phase and the continuous phase, (iii) to increase with increasing the ratio of the radii between the extraction capillary and the nozzle, namely with decreasing confinement, (iv) to increase with the increasing inter-distance between the nozzle and the extraction capillary, namely with decreasing embedment; all these dependencies being related to the viscous forces associated to the droplet detachment, like the gravity force in pendant droplets. On the contrary, the droplet size has been found to be rather insensitive to inertia, to the inclination angle of the nozzle and the inclination angle of the inner diameter of the extraction capillary, even though these parameters have been shown to affect the quasi-static dripping to jetting transition. Remarkably, decreasing confinement or embedment has been numerically shown to displace the transition towards larger values of $Ca_c$ and enhance the dripping regime. This would however have to be confirmed with future experiments.

Besides the excellent agreement obtained between experiments and simulations, not only in the quasi-static limit but also in transient, we have experimentally identified the dripping to jetting transition when increasing the dispersed flow rate ($Q_d$) for a given continuous flow rate ($Q_c$). This transition has however not been explored in detail using our simulations as it requires full transient parametric analysis, hence much more computing resources than for simulating steady flows. Another justification for having disregarded a full parametric analysis in transient for the dripping regime is that the experimental droplet size has been shown to vary only slightly with $Q_d$ at a fixed $Q_c$. This characteristic is remarkable as it demonstrates the dominant role of the viscous forces induced by the continuous phase on the droplet formation and it therefore supports the pertinence of the quasi-static approach beyond its validity domain. Consequently, since increasing $Q_d$ has little effect on the droplet size, it essentially modulates the droplet frequency (see Fig. 3).

**Figure 14:**
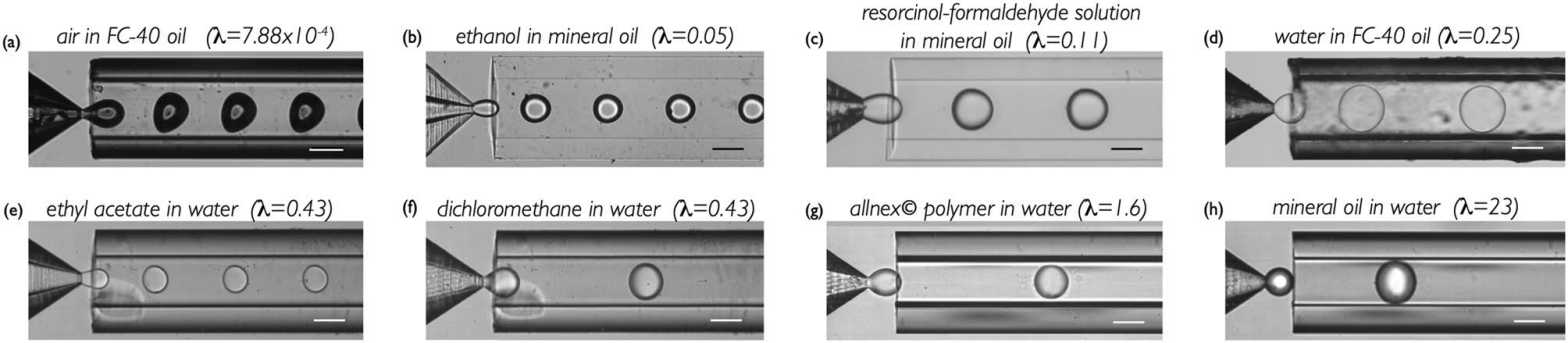
Experimental images of droplet generation in the dripping regime using the Raydrop with different couples and various pairs of fluids in a wide range of viscosity ratios. The scale bar is 100 $\upmu$m.

While this study has been performed for water in oil, we illustrate in Fig. 14 the generation of bubbles and droplets involving a wide variety of pairs of fluids.

This figure demonstrates the universality of the Raydrop to operate properly independently of the wetting properties of the materials in contact with the fluids, and independently of the physico-chemical properties of these fluids (interfacial tension, viscosity, density, miscibility). It additionally indicates that tuning the diameters of the nozzle tip and/or the extraction capillary enables to cover a wide range of droplet diameters, theoretically from 20 to 400$\,\upmu$m, with any given couple of fluids, a feature which is not achievable today with any other single device available.

In the context of a growing demand of controlled droplets in many areas, the Raydrop emerges therefore as a very robust and versatile solution easily implementable in laboratories with little experience and facilities in microfluidics.

## Supplementary information

Supplementary information.
