# Supplementary material for: Microfluidic droplet generation based on non-embedded co-flow-focusing using 3D printed nozzle
Source: Sci Rep. 2020 Dec 10;10:21616. doi: 10.1038/s41598-020-77836-y (PMC7729985; doi:10.1038/s41598-020-77836-y)
Supplement: Supplementary file 1 — Supplementary information. [file 41598_2020_77836_MOESM1_ESM.pdf]

Supplementary Information for
“Enhancement of microfluidic dripping regime by non-embedded co-flow-focusing using 3D printed nozzle”

Adrien Dewandre[1], Javier Rivero-Rodriguez[1], Youen Vitry[1], Benjamin Sobac[1] and Benoit Scheid[1]
[1] *TIPs Lab, Université libre de Bruxelles, Brussels, Belgium*

Correlation functions for varying viscosity ratio

Figure S1(a) plots $Ca_{c,0}$ and $Ca_{c,\infty}$ relating the equivalent quasi-static droplet radius $\bar{R}_m^D$ with the capillary number of the continuous phase in the limits of zero and infinite viscosity ratio λ. Figure S1(b) shows the correlation function λ_* involved in eqn (13) of the manuscript describing the influence of λ on Ca_c in the quasi-static dripping regime for the geometry of couple 1.

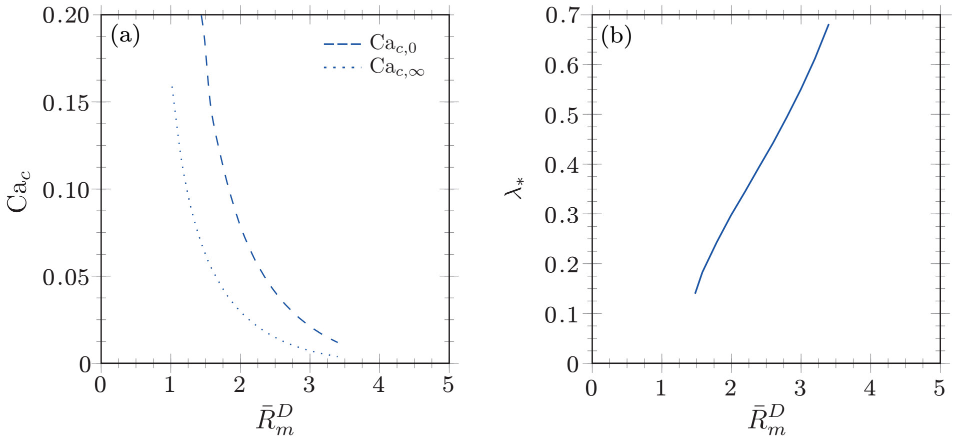

FIG. S1: Computed functions involved in eqn (13), which describes the influence of λ on Ca_c in the quasi-static dripping regime for the geometry of couple 1: (a) $\mathrm{Ca}_{c,0}$, $\mathrm{Ca}_{c,\infty}$ and (b) λ_*. Note that the $\mathrm{Ca}_{c,0}$ (dashed line) is identical to the red curve in Fig. 10 of the manuscript.

Coefficient of Variation (CV) for $\bar{H} \leq 0$

Figure S2 shows the influence of the inter-distance between the nozzle and the extraction capillary on the dripping-jetting transition in the quasi-static regime. This transition becomes independent on $\bar{H}$ for $\bar{H} \leq 0$, i.e. when the injection capillary is embedded into the extraction capillary, as for a simple co-flow configuration (see Fig. 1(b) of the manuscript). This observation is in good agreement with the prediction of the numerical model in Fig. 12(b) of the manuscript.

Tip-multi-breaking for $\bar{H} > 4$

As shown in Fig. S3, we experimentally observed for $\bar{H} > 4$, an other regime of droplet formation in the quasi-static regime, characterized by a periodic succession of identical groups of droplets with decreasing diameters. All other things being equal, the number of droplets in each group increases with $\bar{H}$. This regime is called *tip-multi-breaking* and has been obtained by Zhu et al. (2015) in an embedded co-flow-focusing as represented in Fig. 1(d) of the manuscript.

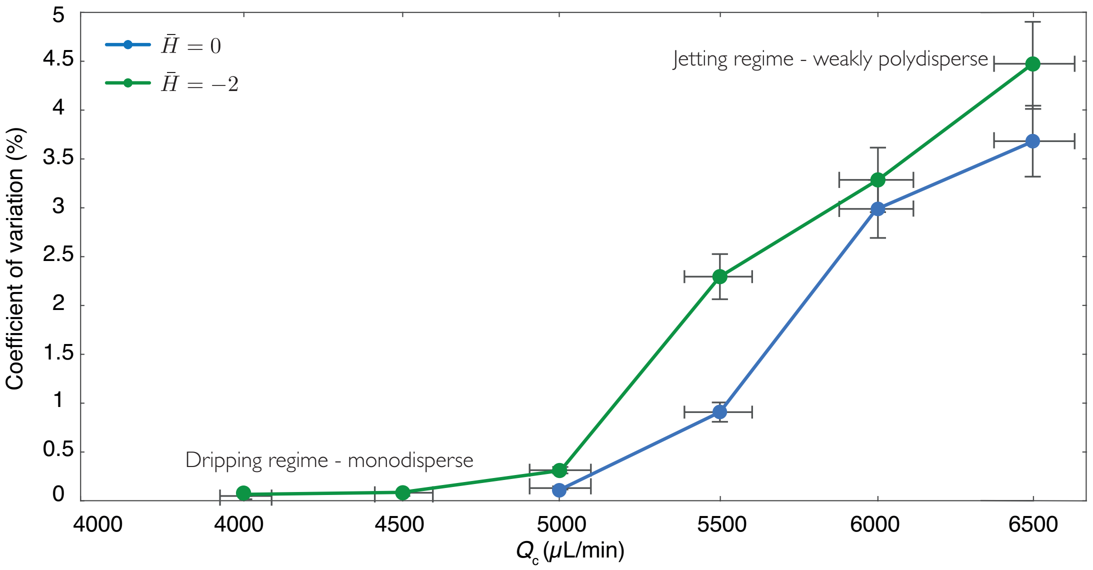

FIG. S2: Coefficient of variation (CV) versus Q_c for Q_d fixed at 50 μL/min and two values of $\bar{H}$ in the couple 2 configuration. The transition between the dripping monodisperse regime and the weakly polydisperse jetting regime occurs for $Q_c \approx 5200 \pm 500$ μL/min.

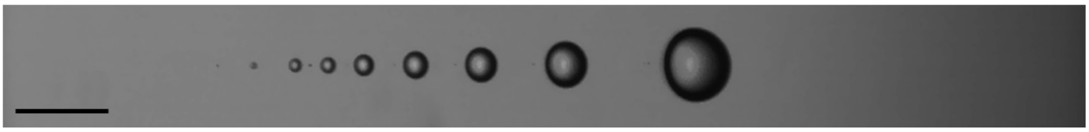

FIG. S3: Tip-multi-breaking regime obtained in a Raydrop with the couple 2 for $\bar{H} = 6$, $Q_c = 5000$ μL/min and $Q_d = 50\mu$L/min. The bar scale is 100 μm.

Influence of the opening angle of the extraction capillary

We show in Fig. S4 that opening the inner diameter of the extraction capillary with a given angle β can displace the quasi-static dripping to jetting transition (represented by crosses in the right plot) to larger values of Ca_c, as already reported in planar flow-focusing by Tan *et al.* (2006).

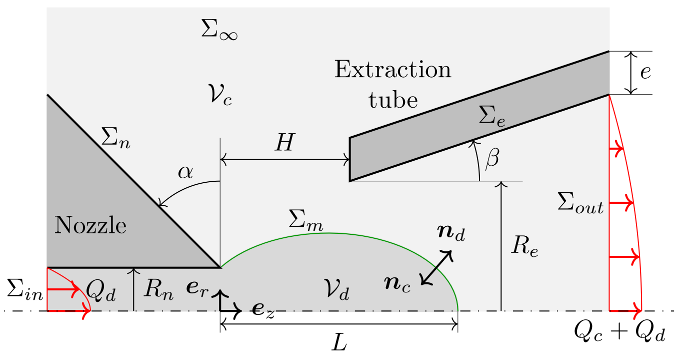

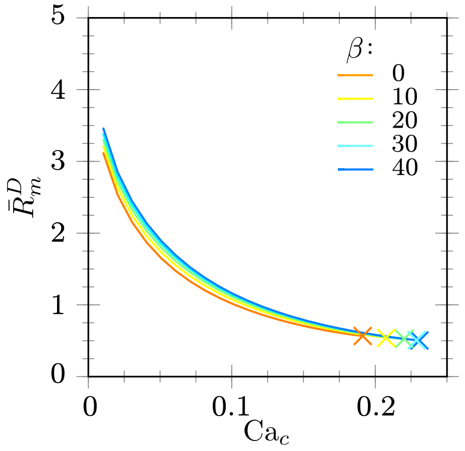

FIG. S4: (Left) Sketch of the droplet generation inside a Raydrop with angle $\beta > 0$. (Right) Influence of β on the quasi-static dripping-jetting transition (crosses).
